# A Comprehensive Study of Xenon Anesthesia in Patients with Locally Advanced Gastric Cancer: A Single-Center Study

**DOI:** 10.3390/medsci14010146

**Published:** 2026-03-18

**Authors:** Natalia Yunusova, Vladimir Faltin, Dmitry Svarovsky, Olga Cheremisina, Elena E. Sereda, Alexandra Augustinovich, Evgeny Usynin, Marina Stakheyeva, Gelena Kakurina, Marina Vusik, Natalia Popova, Viktoria Velikaya, Sergey Afanasiev

**Affiliations:** 1 Cancer Research Institute of the Tomsk National Research Medical Center of the Russian Academy of Sciences, Kooperativny str., 5, Tomsk 634009, Russia; faltin.vladimir@yandex.ru (V.F.); svarovsky.d.a@gmail.com (D.S.); cheremisinaov@oncology.tomsk.ru (O.C.); shashova7ssmu@gmail.com (E.E.S.); aov862@yandex.ru (A.A.); gusi70@list.ru (E.U.); stakheyevam@oncology.tomsk.ru (M.S.); kakurinagv@oncology.tomsk.ru (G.K.); vusikmv@oncology.tomsk.ru (M.V.); popova75tomsk@mail.ru (N.P.); viktoria.v.v@inbox.ru (V.V.); 2 Department of Biochemistry and Molecular Biology with the Course of Clinical Laboratory Diagnostics, Siberian State Medical University, Moskovsky Tract, 2, Tomsk 634050, Russia

**Keywords:** gastric cancer, anesthesia, xenon, dexmedetomidine, sevoflurane, tromboelastometry, Clavien-Dindo grading, postoperative pain syndrome

## Abstract

**Objective:** The objective of this study was to choose the optimal anesthesia method for gastric cancer patients undergoing surgery with lymph node dissection. **Materials and Methods:** The study included 53 patients with stage T1-4aN0-3M0 gastric cancer, who underwent radical surgery with xenon and dexmedetomidine (DMM) anesthesia in combination with epidural analgesia (main group, 27 patients) or with sevorflurane anesthesia in combination with epidural analgesia (comparison group, 26 patients). All patients underwent monitoring of hemodynamic parameters, blood coagulation system, thromboelastometry, and inflammation and metabolic parameters (interleukins, hormones and glucose levels), with an assessment of complications according to the Clavien-Dindo classification and the intensity of postoperative pain. **Results:** Awakening and extubation times, narcotic analgesic consumption, and Numeric Rating Scale pain scores were lower in the xenon + DMM group than in the sevoflurane group (*p* < 0.05). The overall number of patients experiencing complications did not differ significantly between anesthesia types; however, significant differences were found in the total number of complications (*p* = 0.003), the number of complications according to Clavien-Dindo I (*p* = 0.043) and II (*p* = 0.019), and the incidence of postoperative nausea and vomiting (*p* = 0.042). **Conclusions:** The BIS monitoring data obtained showed a sufficient level of anesthesia depth during surgery in both groups; however, post-anesthesia depression persisted longer in patients in sevoflurane group. Mathematical models for predicting Clavien-Dindo IIIb-V complications and severe postoperative pain syndrome are characterized by high sensitivity and specificity. They include simple clinical and laboratory parameters as well as type of anesthesia as predictors. The limitations of predictive models are also discussed in the article.

## 1. Introduction

Surgery triggers a complex stress response involving not only tissue trauma, but also humoral changes, leading to increased levels of cortisol, catecholamines, corticotropic hormones, and insulin. However, the generalization of these changes and their excessive severity with the suppression of autoregulatory mechanisms can become an independent pathogenetic mechanism, aggravating the course of the intra- and postoperative periods and leading to severe complications like thrombosis. Increased coagulation, vascular wall damage, and resulting microthrombosis exacerbate microvascular dysfunction, leading to tissue ischemia and metabolic distress. This results in a “vicious circle” that promotes the progression of organ and system failure, which is a prerequisite for the development of multiple organ failure, which is currently the most common cause of death in intensive care units [1,2,3,4]. In cancer patients, early tumor growth induces systemic homeostasis disruption, characterized by an inflammatory response, chronic disease anemia (often with functional iron deficiency), and endogenous intoxication. All these conditions are accompanied by hemodynamic and hemorheological shifts, oxidative stress, and mitochondrial dysfunction, resulting in decreased tissue perfusion, and intracellular hypoxia and acidosis [4,5,6].

Current anesthesia techniques cannot entirely eliminate physiological disruptions to bodily systems during surgery and postoperatively. The use of xenon as an anesthetic gas in various surgeries, including oncology, has demonstrated significant advantages over other inhalation and non-inhalation anesthetics. Additionally, xenon inhibits N-methyl-D-aspartate (NMDA) receptors in the central nervous system, competing with glycine at its binding site. Blocking NMDA receptors prevents the influx of Ca^2+^ and Na^+^, causing an anesthetic effect. Xenon also activates TREK-1, TASK-3, and KATP channels, leading to K^+^ efflux and a neuroprotective effect. Xenon anesthesia is reported to have antiemetic, anti-inflammatory, immunomodulatory, neuroprotective, cardioprotective and analgesic effects, alongside excellent hemodynamic stability [7,8,9,10,11,12,13,14]. However, it may not provide sufficient depth for major abdominal surgery when used as a monoanesthetic. Dexmedetomidine (DMM), a highly selective alpha-2 agonist, has recently attracted considerable interest from anesthesiologists. It is used for sedation in intensive care settings and is increasingly being used in anesthesiology [15,16,17]. The aim of the study was to evaluate the efficacy of xenon + DMM anesthesia combined with epidural analgesia in radical gastric cancer surgery, identifying perioperative markers of postoperative complications and pain intensity.

## 2. Materials and Methods

### 2.1. Patients

Study design suggested a prospective cohort-like comparison of two anesthetic strategies. The study included 53 patients with histologically confirmed gastric cancer (T1-4aN0-3M0), who underwent radical surgery. The patients were divided into two groups depending on the anesthesia technique. The main group consisted of 27 patients who received anesthesia with xenon and DMM combined with epidural analgesia. The comparison group consisted of 26 patients who received anesthesia with sevorflurane combined with epidural analgesia. The patients were enrolled into the study groups consecutively and prospectively, independent of surgeon or anesthesiologist preference. Clinical characteristics, such as tumor location and size were used only to determine whether subtotal or total gastrectomy was performed, while the anesthetic technique and perioperative care protocols remained consistent regardless of disease factors. Factors such as age, degree of anesthetic risk (ASA Physical Status Classification), baseline severity of comorbidities, tumor stage, surgery duration, and blood loss were used as inclusion and exclusion criteria. On average, intraoperative blood loss in patients in the compared groups did not exceed 200–300 mL. Indications for intraoperative blood transfusion of any volume were exclusion criteria for patients from the study.

The clinical and morphological characteristics of the patients in the study groups are presented in Table 1. The intensity of postoperative pain was assessed using a Numeric Rating Scale (NRS) in accordance with the recommendations for use of the Common Terminology Criteria for Adverse Events (version 5.0, 2017) of the US National Cancer Institute [18]. Incidence of postoperative complications according to the Clavien-Dindo grading was also evaluated [19].

### 2.2. Anesthesiology Technique and Intraoperative Monitoring

After admission to the operating room and establishment of venous access, intravenous administration of DMM was started at a dose of 0.6–0.8 mcg/kg/h. Then, an epidural puncture was performed at the T7–T9 level, after which an infusion of an analgesic mixture was started: 47 mL of 0.2% ropivacaine; 2 mL of 0.005% fentanyl; 0.1 mL of 0.1% adrenaline. Standard premedication was then administered, followed by induction with propofol 2–2.5 mg/kg, and muscle relaxation with rocuronium bromide 0.6 mg/kg. During the denitrogenation period, sedation was administered only with DMM. Xenon was then injected into the closed circuit of the anesthesia device Axeoma TM (Alfa-Impex Oy, Helsinki, Finland). Once a stable equilibrium of 60:40 Xe: O_2_ was achieved, low-flow anesthesia was administered. DMM administration was continued throughout the surgery under hemodynamic monitoring, with the DMM dosage varied within the range of 0.3–0.6 mcg/kg/h. DMM administration was stopped 20 min before the end of surgery. DMM administration was not performed in the early postoperative period. For postoperative analgesia, both study groups continued to administer an analgesic mixture (47 mL of 0.2% ropivacaine; 2 mL of 0.005% fentanyl; 0.1 mL of 0.1% epinephrine) via the epidural catheter at a rate of 5–10 mL/hour during the first 24 h of the postoperative period. Intravenous bolus administration of 0.1 mg fentanyl was administered intraoperatively during tumor mobilization and lymph node dissection in all patients.

Patients from the comparison group received standard low-flow anesthesia with sevoflurane combined with epidural analgesia without DMM administration. Patients of both groups, who experienced postoperative nausea and vomiting were given ondansetron 8 mg intravenously. All patients underwent intraoperative monitoring. The parameters presented in the tables and figures were measured at the following points: Point 1—patient condition in the morning before surgery; Point 2—the traumatic stage of the surgery (mobilization of the macrospecimen); Point 3—after the patient’s extubation. The following hemodynamic parameters were assessed using the esCCO technology: systolic, diastolic, and mean blood pressure, heart rate, eCCO (estimated continuous cardiac output), eCI (estimated cardiac index), eSV (estimated stroke volume), eSI (estimated stroke index), and arterial hemoglobin oxygen saturation (SpO2). The depth of anesthesia was assessed using BIS monitoring parameters.

### 2.3. Hormones, Acute Phase Protein, Cytokine, and Glucose Levels

Cytokines (IL-1β, -6, -8, -10, TNF-α), C-reactive protein, and cortisol concentrations were determined using Vector-Best enzyme-linked immunosorbent assays (Vector-Best, Novosibirsk, Russia), insulin was measured using the Insulin ELISA kit (DRG, Marburg, Germany), and adrenocorticotropic hormone (ACTH) was measured using the Biomerica ACTH ELISE kit (Irvine, CA, USA). Blood glucose levels were measured using an Accu-Chek glucometer (Roche Diabetes Care GmbH, Mannheim, Germany).

### 2.4. Evaluation of the Blood Coagulation System and Thromboelastometry Parameters

The potential impact of the anesthetic method on the hemostatic system was assessed by measuring prothrombin time, activated partial thromboplastin time (APTT), thrombin time, soluble fibrin-monomer complexes (SFMC), and fibrinogen. The following parameters were determined by rotational thromboelastometry using a ROTEM delta (PENTAPHARM GmbH, Munich, Germany) device: CT—the clot formation time required to reach an amplitude of 2 mm, characterizes the initial stage (initiation) of blood coagulation, thrombin formation; CFT—clot formation time (the time interval between reaching 2- and 20-mm amplitudes), characterizes fibrin polymerization under the influence of coagulation factor XIII and platelets; α-angle, characterizing the kinetics of clot formation; A(10) is the clot hardness after 10 min, characterizing the density and stability of the clot at the specified time; MCF is the maximum clot hardness (a measure of clot hardness, which represents the maximum amplitude reached by the time the fibrinolysis process begins to cause its decrease); MCE is the maximum flexibility (elasticity) of the clot, reflecting its mechanical properties. This parameter is calculated using the formula MCE = MCF × 100/(100 − MCF).

In the EXTEM test, after the startem reagent, the ex-TEM reagent containing phospholipids; tissue factor, which initiates coagulation via the extrinsic pathway; and heparinase, which neutralizes the possible effects of heparin present in the blood, were added to the blood sample. In the INTEM test, the in-TEM reagent containing phospholipids from rabbit brain and ellagic acid, which initiate the intrinsic pathway, were added to the blood sample. The parameters described in Section 2.3 and Section 2.4 were measured as follows: 1st point—the day before the operation, in the morning on an empty stomach; 2nd point—during the operation at the stage of mobilization of the macropreparation; 3rd point—after extubation of the patient; 4th point—early postoperative period (first day).

### 2.5. Statistics

**Figure 1:**
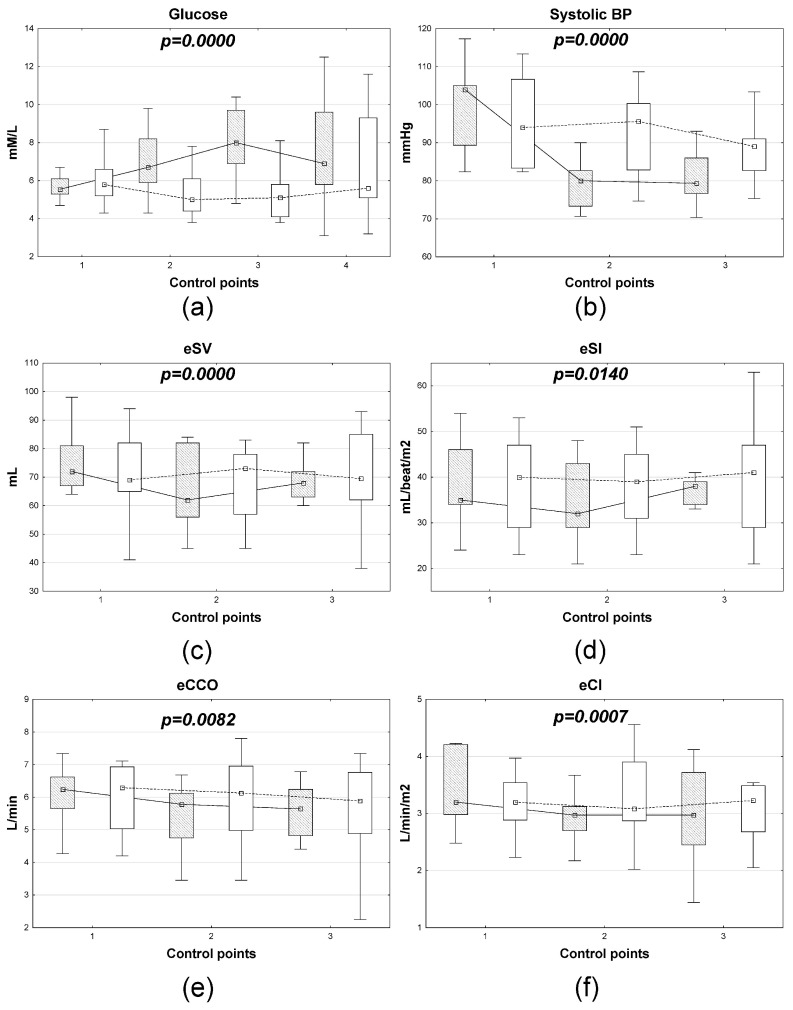
Dynamics of changes in plasma glucose concentration (**a**) and hemodynamic parameters (**b**–**f**) in patients with gastric cancer depending on the type of anesthesia. Note: open boxes represent xenon + DMM, shaded boxes represent sevoflurane, the *x*-axis represents control points, and the *y*-axis represents marker levels. Boxplots: represent the distribution of the data, where the box indicates the interquartile range (25th–75th percentiles), the central point: represents a measure of central tendency, and the whiskers: indicate the range of the data (minimum to maximum values); different hatch patterns correspond to distinct groups. The lambda coefficient value for each discriminant model is shown in the corresponding blocks of Figure 1.

**Figure 2:**
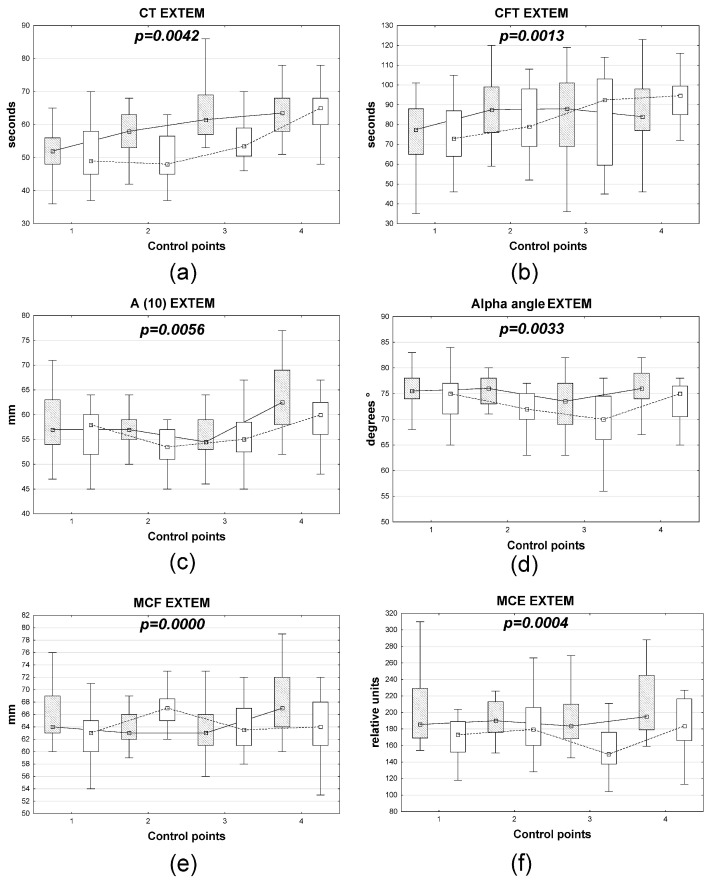
Dynamics of changes in EXTEM rotational thromboelastometry parameters (**a**–**f**) in patients with gastric cancer depending on the type of anesthesia. Note: open boxes represent xenon + DMM, shaded boxes represent sevoflurane. The *x*-axis represents control points, and the *y*-axis represents marker levels. Boxplots represent the distribution of the data, where the box indicates the interquartile range (25th–75th percentiles), the central point represents a measure of central tendency, and the whiskers indicate the range of the data (minimum to maximum values); different hatch patterns correspond to distinct groups.The lambda coefficient value for each discriminant model is shown in the corresponding sections of Figure 2.

**Figure 3:**
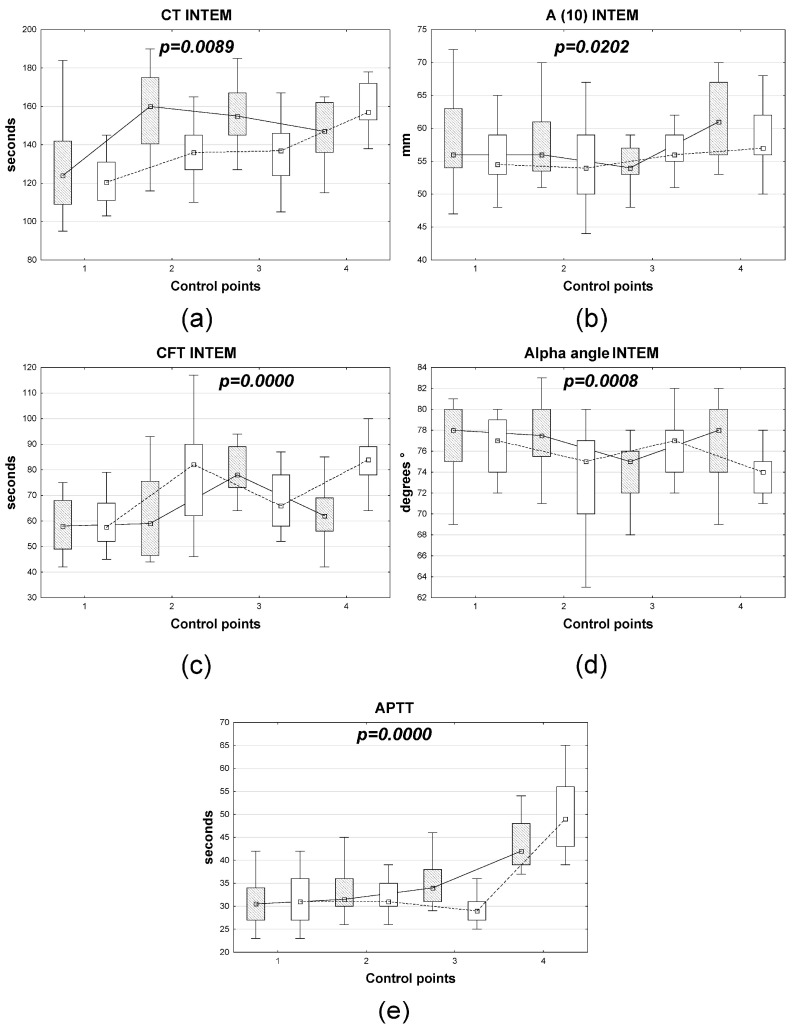
Dynamics of changes in INTEM rotational thromboelastometry (**a**–**d**) and coagulation system parameters (**e**) in patients with gastric cancer depending on the anesthesia option. Note: open boxes represent xenon, shaded boxes represent sevoflurane, the *x*-axis represents control points, and the *y*-axis represents marker levels. Boxplots: represent the distribution of the data, where the box indicates the interquartile range (25th–75th percentiles), the central point: represents a measure of central tendency, and the whiskers: indicate the range of the data (minimum to maximum values); different hatch patterns correspond to distinct groups. The lambda coefficient value for each discriminant model is shown in the corresponding blocks of Figure 3.

Statistical analysis was performed using Statistica 10.0 software. For all analyses, differences were considered statistically significant at a *p* < 0.05 level. Data in tables and graphs are presented as medians and interquartile ranges or as M ± m. The significance of differences in the studied data was tested using nonparametric tests: the Mann–Whitney U test (pairwise comparisons of independent sets of indicators), the Wilcoxon W test (pairwise comparisons of dependent sets of indicators), and Friedman’s repeated measures analysis (for related samples). The Chi-Square test with Yates’ correction was used to assess the significance of differences for categorical variables, using statistical tables in the Clinical Research Calculation module (http://vassarstats.net, accessed on 10 March 2026). Discriminant analysis was used to assess the significance of differences in the dynamics of measured parameters in the study groups. Figure 1, Figure 2 and Figure 3 show the significance level (*p*) of the Wilks’ lambda test for the discriminant model. Stepwise logistic regression was used to construct the predictive models. The sensitivity and specificity of the models were assessed using ROC analysis. The parameters of the resulting model were tested using the jackknife method.

The multiple primary endpoints in our study were the incidence of complications Clavien-Dindo IIIb-V, as well as the incidence of severe pain syndrome in the early postoperative period in the compared groups. The secondary endpoint was the incidence of complications Clavien-Dindo I-IIIa. When analyzing the exploratory endpoints (marker levels, BIS monitoring, thromboelastometry and hemodynamic parameters, etc.), adjustment for multiple comparisons was not used.

## 3. Results

### 3.1. Comparison of Changes in Inflammatory Markers, Stress and Metabolic Hormones, Cytokines, and Plasma Glucose in Gastric Cancer Patients with Different Anesthetic Strategies

**Figure 4:**
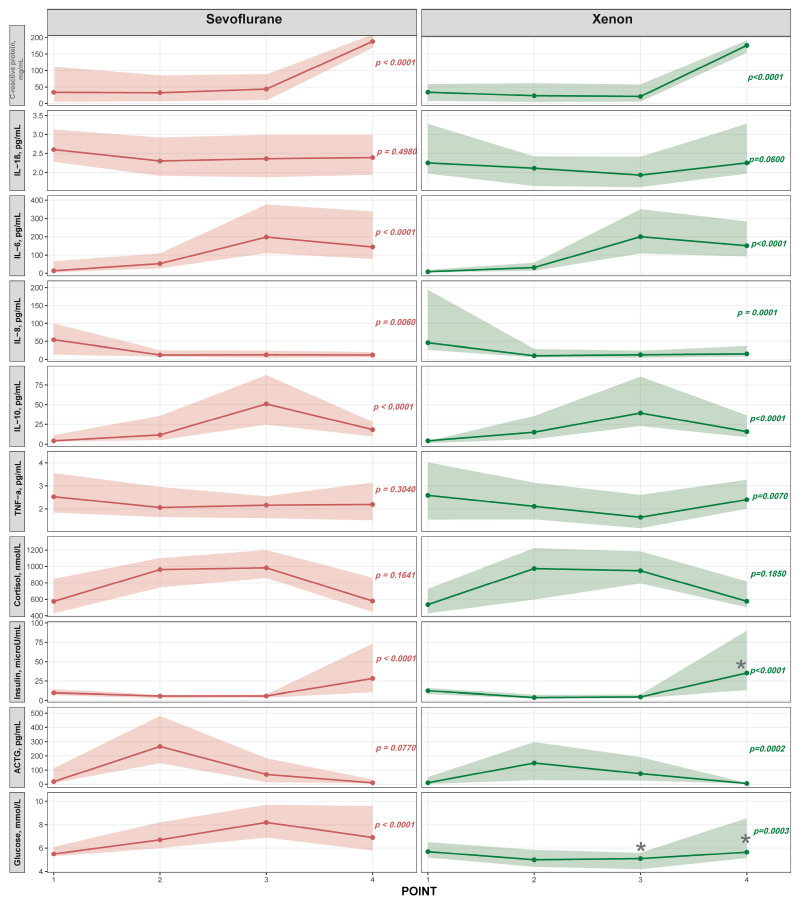
Changes in inflammatory, hormonal, and metabolic parameters during anesthesia with sevoflurane and xenon + DMM. Temporal changes in C-reactive protein, pro- and anti-inflammatory cytokines (IL-1β, IL-6, IL-8, IL-10, TNF-α), cortisol, insulin, ACTH, and glucose across four measurement points (1–4) in patients receiving sevoflurane (left panels) or xenon + DMM (right panels). Data are presented as median values (solid lines with points) with interquartile ranges (shaded areas, Q1–Q3). Identical *y*-axis scales are used for direct comparison between anesthetic modalities within each parameter. *p*-values (Friedman test) indicate the statistical significance of within-group temporal changes and are shown in the corresponding color of the anesthetic agent. Note: *—differences between groups (Mann-Whitney test).

Overall, the changes in inflammatory markers, stress and metabolic hormones, and glucose levels during the surgical stages were similar in gastric cancer patients with any anesthesia strategies. Differences were primarily in the rate of marker increases or decreases during the surgical stages (Figure 4). Both groups showed a significant increase in C-reactive protein and IL-6 levels by the 4th checkpoint, as well as a significant rise in ACTH levels at the 2nd checkpoint in the xenon group, while IL-8 levels progressively decreased by the 4th checkpoint in both groups. No significant changes were noted for IL-1β and cortisol in either group, and no significant changes were observed for TNFα in the sevoflurane group. Both types of anesthesia were accompanied by a decrease in insulin levels at points 2 and 3, followed by a sharp rise in plasma insulin at point 4. In the xenon subgroup, hyperinsulinemia on the first day after surgery was more pronounced compared to sevoflurane anesthesia (*p* < 0.05).

### 3.2. Comparison of Hemodynamic, Coagulation, and Thromboelastometry Parameters in Gastric Cancer Patients with Different Anesthetic Techniques

According to discriminant analysis, the dynamics of the following parameters statistically differed significantly between gastric cancer patients with different combined anesthesia options: serum glucose, systolic BP, eSV, eSI, eCCO, and eCI. Furthermore, fundamentally different dynamics were observed for several parameters (glucose, mean BP, eSV, eSI, and eCI). For eCCO, a gradual decrease in varying severity was noted by the first day after surgery in both groups (Figure 1).

When performing ROTEM/EXTEM (Figure 2), an increase in the CT parameter by the 4th control point was observed in the xenon + DMM anesthesia group, along with a wave-like dynamics of the APTT, A10, MCE, MCF, and α angle during the surgical treatment stages. The EXTEM CFT, which characterizes maximum clot hardness—that is, the maximum amplitude reached by the time fibrinolysis begins to cause its decline—had fundamentally different dynamics during the perioperative period under different anesthesia strategies. CFT gradually increased during the first postoperative day in patients anesthetized with xenon + DMM (*p* < 0.05 according to the Friedman test), while in patients in the sevoflurane group, this indicator was the same preoperatively and on the first postoperative day, and during surgery, a 29% increase was observed (*p* < 0.05 according to the Friedman test).

According to discriminant analysis, the dynamics of the following coagulation system parameters and ROTEM/INTEM thromboelastometry statistically significantly differed between gastric cancer patients with different anesthesia options: APTT, CT, A10, CFT, and alpha angle. Furthermore, fundamentally different dynamics were observed for a number of parameters (APTT, CT, CFT, and alpha angle). Meanwhile, for A10, the dynamics of the parameter were consistent across groups, but with varying levels of decline or increase (Figure 3).

### 3.3. Perioperative Period Characteristics After Gastric Cancer Surgery with Different Anesthesia Strategies

Perioperative period characteristics are presented in Table 2.

BIS monitoring data demonstrated a sufficient level of anesthesia depth during surgery in both study groups (Table 2), but it should be noted that postanesthesia depression symptoms persisted longer in patients in the control group after extubation (*p* < 0.05). The awakening and extubation times were shorter in patients in the study group compared to the control group (*p* < 0.05). Statistically significant differences in pain severity, assessed using the NRS, were revealed between the groups, which was accompanied by a reduction in the amount of opioid analgesics used in the postoperative period, in particular fentanyl (Table 2).

### 3.4. Incidence of Postoperative Complications

The incidence of postoperative complications according to the Clavien-Dindo grading is presented in Table 3.

A total of 87 complications were recorded in 53 patients. The groups included patients who developed multiple complications at various points postoperatively. The complications had varying degrees of severity according to the Clavien-Dindo grading. Uncomplicated course was detected in 10 patients (37%) of the xenon + DMM anesthesia group, while in the sevoflurane anesthesia group, uncomplicated course was observed in 1 patient (3.9%) (*p* = 0.0111). Evaluation of early postoperative complications showed that in the xenon group, complications were recorded in 17 (63.9%) patients, a total of 18 complications were identified, and multiple complications were detected in 1 patient (3.7%). In the sevoflurane group, complications were observed in 25 (96.2%) patients, 59 complications were recorded, and the overwhelming majority—23 patients (88.0%)—from this group had multiple complications. Although, in general, no statistically significant differences were found in the number of patients with complications between the groups with different strategies of anesthesia, significant differences were found in the total number of complications (*p* = 0.003), the number of complications according to Clavien-Dindo I (*p* = 0.043) and II (*p* = 0.019), and the incidence of postoperative nausea and vomiting (*p* = 0.042) (Table 3).

### 3.5. Predicting the Incidence of Complications According to Clavien-Dindo IIIb-V and the Severe Pain Syndrome in Patients with Gastric Cancer

Given the limited number of outcome events, predictive modeling was performed as an exploratory analysis. Patients with severe complications according to the Clavien-Dindo IIIb-V classification represent a particular medical and social challenge. An analysis conducted on a sample of 53 patients with gastric cancer who underwent radical surgery showed that the type of anesthesia is not an independent statistically significant factor influencing the incidence of Clavien-Dindo IIIb-V complications. Maintaining the highest possible quality of life is also an important component of antitumor therapy. Therefore, the goal of this block of study was to develop mathematical models for predicting the incidence of complications according to Clavien-Dindo IIIb-V and the severity of pain in patients with gastric cancer. Predictors included in the analysis included clinical and morphological parameters, anesthetic risk scores according to the ASA scale, type of anesthesia, hormonal and metabolic parameters, interleukin and proinflammatory marker levels, hemodynamic parameters, traditional coagulation profile, and EXTEM and IMTEM thromboelastometry indicators, assessed before surgery (variant 1). In the second variants, predictors included clinical and morphological parameters, hormonal and metabolic parameters, interleukin and proinflammatory marker levels, hemodynamic parameters, traditional coagulation profile, and EXTEM and IMTEM thromboelastometry indicators, assessed immediately after patient extubation (variant 2). When predicting the probability of developing Clavien-Dindo IIIb-V complications in the postoperative period, the regression equation was

F = −24.112 + 1.793*[type of anesthesia] − 0.1*[sAP] + 0.144*[CT ex] + 0.291*[alpha angle INTEM],

where −24.112 is a constant; [type of anesthesia] is the type of anesthesia: 1-xenon + DMM, 2-sevoflurane; [sAP] is the systolic arterial pressure, mmHg; [CT EXTEM] is the EXTEM thromboelastogram value, s; and [Alpha angle INTEM] is the INTEM thromboelastogram value (variant 1).

Next, the probability of developing Clavien-Dindo IIIb-V complications was calculated using the formula

P = 1/(1 + e^(−F)),

where P is the probability of developing complications, e (base of the natural logarithm) = 2.718, and F is the regression function value. A *p* value ≥ 0.5 indicates a high probability of developing Clavien-Dindo IIIb-V complications, while a *p* value < 0.5 indicates a high probability of an uncomplicated postoperative course or complications of Clavien-Dindo I-IIIa. The sensitivity and specificity estimates for this model were 70% and 96%, respectively. ROC analysis demonstrated limited discriminative ability of the model, with an AUROC of 0.53 (95% CI 0.35–0.71). The model characteristics are presented in Table 4.

When predicting the likelihood of developing severe postoperative pain syndrome (NRS 8–10 points), the regression model had the following form:

F = 33.422 + 4.961*[type of anesthesia] − 4.373*[glucose] + 0.145*[CFT EXTEM] + 0.129*[MCF EXTEM] − 0.136*[A(10) INTEM] − 0.43*[Alpha angle INTEM],

where 33.422 is a constant; [type of anesthesia] is the type of anesthesia: 1-xenon + DMM, 2-sevoflurane; [glucose] is the concentration of glucose in the blood, mmol/L; [CFT EXTEM], [MCF EXTEM] are the thromboelastogram parameters in the EXTEM variant; and [A (10) INTEM] and [Alpha angle INTEM] are the thromboelastogram parameters in the INTEM variant. The sensitivity and specificity estimates for this model were 87% and 100%, respectively. ROC analysis demonstrated an AUROC of 0.86 (95% CI 0.75–1.00). Given the very limited number of severe pain events (*n* = 6), the model is likely overfitted and should be interpreted with caution. Model characteristics are presented in Table 5.

The probability of postoperative complications IIIb-V on the Clavien-Dindo scale (variant 2) was calculated using the formula

P = 1/(1 + e∧(−F)),

where

F = −5.216 + 0.874*[type of anesthesia] − 0.2*[sBP] − 0.1*[CFT EXTEM] + 0.037*[CT INTEM],

where −5.216 is a constant; [type of anesthesia] is the type of anesthesia: 1-xenon + DMM, 2-sevoflurane; [sBP] is systolic blood pressure; [CFT EXTEM] is the EXTEM thromboelastography value; and [CT INTEM] is the INTEM thromboelastography value. The sensitivity and specificity of the model were 78.1% and 75%, respectively. ROC analysis showed moderate discriminative performance, with an AUROC of 0.60 (95% CI 0.42–0.78). The characteristics of the model variables are presented in Table 6.

The probability of severe pain syndrome development (variant 2) is calculated using the formula

P = 1/(1 + e∧(−F)),

where

F = −2.213 + 0.514*[anesthesia type] + 0.487*[glucose] − 0.107*[A (10) in] + 0.349*[SFMC],

where −2.213 is a constant; [anesthesia type] is the type of anesthesia: 1-xenon + DMM, 2-sevoflurane; [glucose] is the blood glucose concentration, mmol/L; [A (10) in] is the INTEM thromboelastography index; and [SFMC] are soluble fibrin–monomer complexes, seconds. The sensitivity and specificity of the model were 86% and 100%, respectively. ROC analysis showed an AUROC of 0.81 (95% CI 0.47–1.00), indicating moderate discriminative performance within this cohort. The model characteristics are presented in Table 7.

## 4. Discussion

Xenon and DMM create a highly stable, synergistic anesthetic combination that provides excellent hemodynamic stability [12,15,16,17]. BIS monitoring data showed a sufficient level of anesthesia depth during surgery in both study groups, but post-anesthesia depression symptoms persisted longer in patients in the control group. Our results are consistent with data from other researchers, which indicate a favorable early perioperative period, for example, in non-cancer patients aged over 20 years (earlier extubation, eye opening, time/space orientation) [15,20,21]. The receptor structures involved in the analgesic effects of DMM and xenon are not opiate receptors. These effects are mediated through NMDA receptors, and to a lesser extent, other receptors—such as α-amino-3-hydroxy-5-methyl-4-isoxazolepropionic acid (AMPA) receptors, ionotropic glutamate receptors, kainate receptors, and α2-adrenergic receptors—of the central and, to some extent, peripheral nervous systems [14,22,23]. In our study, xenon anesthesia combined with DMM was associated with lower postoperative pain scores and reduced opioid consumption compared with sevoflurane anesthesia.

Our study identified certain differences in the impact of anesthesia type on perioperative insulinemia and glycemia. Both types of anesthesia were associated with a decrease in insulin levels at time points 2 and 3, followed by a sharp rise in plasma insulin levels at time point 4. In the xenon + DMM subgroup, hyperinsulinemia on the first day after surgery was more pronounced compared to anesthesia with sevoflurane (*p* < 0.05). Apparently, it is the insulin dynamics that can explain hyperglycemia in the sevorane group at control points 2, 3, and 4, while in patients in the xenon + DMM group, the median glucose levels at all control points were within the reference values. Xenon anesthesia resulted in significantly lower rates of postanesthesia hyperglycemia (38.3%, 10/26) compared to sevoflurane (61.5%, 16/26, *p* < 0.05) (Figure 4). Literature data indicate the absence of a significant effect of all three drugs on the levels of insulin and glucose; however, a number of studies indicate the presence of individual variations in the level of insulinemia and glycemia, especially in patients with impaired glucose tolerance and diabetes mellitus [20,24]. In addition, xenon maintains normal glucose levels, in particular in neurosurgical patients due to a decrease in oxygenation and the rate of glucose consumption by brain cells [25]. Analysis of the laboratory parameters studied revealed that both types of anesthesia resulted in similar dynamics of proinflammatory markers, cytokines, and hormones, with the exception of insulin levels. This may indicate a similar effect of anesthetics on the systemic inflammatory response syndrome and the hypothalamic-pituitary system as a whole [26]. Both types of anesthesia demonstrated good regulation of central hemodynamic parameters, with all parameters remaining within reference ranges during the perioperative period. However, according to discriminant analysis, the dynamics of the following parameters statistically significantly differed between gastric cancer patients with different anesthesia types: systolic blood pressure, eSV, eSI, eCCO, and eCI. Systolic blood pressure proved to be a significant predictor of severe postoperative complications, and the combination of xenon and DMM appears to offer certain advantages over sevoflurane anesthesia in terms of hemodynamic parameter control.

The analysis of postoperative complications suggests a potential association between xenon + DMM anesthesia and a reduced number of Clavien–Dindo I–IIIa complications in the present study. The antiemetic effect of xenon is achieved by stimulating type III serotonin receptors in the brain [13]. Xenon shows significant promise in reducing neurological injury through neuroprotective properties. Furthermore, a neuroprotective effect has also been demonstrated for DMM (due to central sympatholysis) [7,9,10,11,12,13]. The neuroprotective and antioxidant effects of xenon and DMM, realized at the level of the brain (vomiting and thermoregulation centers), are apparently responsible for the decrease in episodes of vomiting and postoperative hyperthermia in the main study group. Both xenon and DMM have the property of reducing the frequency of postoperative delirium, as well as delirium of other etiologies (for example, opiate delirium, alcoholic delirium) [16,17,20,27]. The mechanism of this action includes a neuroprotective effect realized at various levels of the central and peripheral nervous systems, a reduction in the frequency of hyperglycemic episodes, and a decrease in the probable toxic effects of glucose [9,12,17,20,28]. The lower incidence of pulmonary embolism observed in the xenon + DMM group may be related to differences in perioperative hemodynamic stability and coagulation dynamics reported for this anesthetic regimen both in the literature and in our observations [12,22].

Table 3 presents data on a significant trend towards a decrease in the incidence of postoperative pancreatitis in patients in the xenon + DMM group, which can be explained by the combined effect of both anesthetics, namely, a decrease in the severity of ischemia in critically important organs, including the pancreas, and an improvement in microcirculation in the pancreas due to stable hemodynamics and the absence of capillary spasm. Predicting acute pancreatitis after radical gastric cancer surgery with lymph node dissection is challenging [29,30]. Experts who have been studying this issue for many years recommend implementing preventive measures to prevent pancreatitis not routinely for everyone, but only for high-risk patients [30,31]. Certainly, this approach is probably justified; however, in our study, the use of xenon + DMM anesthesia was associated with a lower incidence of acute pancreatitis compared with sevoflurane anesthesia. This observation may reflect the combined pharmacological effects of these anesthetics, including improved hemodynamic stability and microcirculatory conditions in abdominal organs such as the pancreas.

The lower incidence of complications such as bleeding, acute cerebrovascular accident, anastomotic insufficiency, and multiple organ failure observed in the xenon + DMM group may be related to the complex pharmacological mechanisms of these anesthetics.

Furthermore, experimental and preclinical studies have demonstrated xenon’s immunomodulatory, immunosuppressive, anti-inflammatory, and antioxidant effects. Xenon inhalation reduces neutrophil adhesion to the endothelium by downregulating PSLGL-1 and S-selectin expression, thereby limiting inflammation. Xenon inhalation also suppressed the expression of the Apoe, Trem2, Cd86, Plin2, and Cd14 genes in TE4 and APP/PS1 mice. It is noteworthy that CD14 is an accessory molecule that, together with TLR4, induces NF-κB activation and the induction of proinflammatory genes. Deletion of CD14 is associated with a decrease in the number of microglia and Aβ plaques in APP/PS1 mice [8]. Furthermore, xenon inhalation suppressed inflammatory response and oxidative stress genes in the hippocampus in an APOE mouse model of tauopathy [10]. A certain immunosuppressive and immunomodulatory effect of xenon, characterized by a decrease in neutrophil infiltration in the renal glomeruli, inhibition of inflammasome activation, IgG deposition, and the C3 complement component, was revealed in a model of lupus nephritis [32]. A significant effect of xenon on the anti-inflammatory activation and apoptosis of neutrophils ex vivo was revealed in the work of O.E. [7]. The authors demonstrated that xenon inhalation in volunteers (30% for 60 min) has a pronounced anti-inflammatory effect on neutrophils stimulated with lipopolysaccharides, reducing their activation by inhibiting the pro-inflammatory kinase ERK1/2 and the pro-inflammatory MAP kinase p38, as well as reducing the expression of the activation and degranulation markers CD11b and CD66b on the neutrophil surface. It was shown that stimulation with lipopolysaccharides reduced spontaneous neutrophil apoptosis, while xenon increased the ability of neutrophils to undergo apoptosis, which will likely contribute to the resolution of inflammation. Thus, xenon has a distinct immunomodulatory and antioxidant effect, which can prevent incomplete inflammation, excessive neutrophil activation, and subsequent massive cytolysis, followed by uncontrolled systemic inflammatory response syndrome and multiple organ failure [7].

The mathematical models for predicting severe postoperative complications and severe postoperative pain syndrome are characterized by high sensitivity and, especially, high specificity. They include simple clinical and laboratory parameters as well as type of anesthesia as predictors and can be successfully used in clinical practice. But there are certain limitations to the application of the models. Taken together, the predictive analyses performed in the present study demonstrate limited to moderate discriminative performance within this single-center cohort. A post hoc power analysis indicated that for comparisons of dependent variables (two means, paired t-test) with α = 0.05 and a desired power of 0.9, the required sample size would be at least *n* = 32, whereas analyses of categorical distributions using the χ^2^ criterion would require approximately *n* = 62. The sample size in the present study (*n* = 53) therefore meets the requirements for some analyses but remains slightly below the optimal level for χ^2^-based comparisons, which should be considered when interpreting the results. While certain models showed acceptable AUROC values, confidence intervals were wide, reflecting the relatively small sample size and low number of outcome events. The event-per-variable ratio was suboptimal in several models, increasing the likelihood of coefficient instability and overfitting. Therefore, the proposed prediction equations should be interpreted as exploratory and hypothesis-generating rather than definitive clinical tools. Nevertheless, the analyses provide preliminary insight into potential perioperative factors associated with severe complications and pain intensity, and may serve as a foundation for future, adequately powered studies with external validation.

## 5. Conclusions

The BIS monitoring data demonstrated a sufficient level of anesthesia depth during surgery in both study groups, but postanesthesia depression persisted longer in patients in the control group. The overall number of patients experiencing complications did not differ significantly between anesthesia types; however, significant differences were found in the total number of complications (*p* = 0.003), the number of complications according to Clavien-Dindo I (*p* = 0.043) and II (*p* = 0.019), and the incidence of postoperative nausea and vomiting (*p* = 0.042).

Mathematical models for predicting Clavien-Dindo IIIb-V complications and pain severity are characterized by high sensitivity and, especially, specificity. They include simple clinical and laboratory parameters as predictors and can be used in clinical practice. The authors consider these predictive models exploratory. Further randomized, possibly multicenter, studies are needed, as well as validation of the resulting predictive models in an external validation cohort.

## Figures and Tables

**Table 1:** Clinical and pathological characteristics of patients with gastric cancer.

Parameters	Study Groups		*p*-Level
	Xenon + DMM Anesthesia, *n* = 27 (100%)	Sevoflurane Anesthesia, *n* = 26 (100%)	
Sex:			0.680
Men	13 (48%)	14 (54%)	
Women	14 (52%)	12 (46%)	
Age, years, M ± m	63.3 ± 7.30	63.1 ± 7.50	0.892
Degree of anesthetic risk (ASA Physical Status Classification):			0.985
I	5 (18.5%)	5 (18.5%)	
II	11 (40.7%)	10 (38.5%)	
III	11 (40.7%)	11 (40.7%)	
Stage			0.920
T1-4aN0M0	11 (41%)	10 (38%)	
T3-4aN1-3M0	16 (59%)	16 (62%)	
Histology:			0.036
Adenocarcinoma	11 (41%)	19 (73%)	
Signet ring cell carcinoma	16 (59%)	7 (27%)	
Grade:			0.697
G1	1 (4%)	0	
G2	7 (26%)	5 (19%)	
G3	16 (59%)	18 (69%)	
Unknown	3 (11%)	3 (12%)	
Tumor localization:			0.928
Cardia	9 (33.3%)	10 (38%)	
Body	9 (33.3%)	8 (31%)	
Pylorus	9 (33.3%)	8 (31%)	

**Table 2:** Perioperative period characteristics.

Parameters	Study Groups		*p*-Level
	Xenon + DMM Anesthesia, *n* = 27 (100%)	Sevoflurane Anesthesia, *n* = 26 (100%)	
Duration of surgery, min, M ± m	156 ± 11.6	150 ± 10.7	0.450
Duration of anesthesia, min, M ± m	185 ± 13.9	188 ± 14.5	0.566
Time to regain consciousness, min, M ± m	6.12 ± 1.01	8.56 ± 1.17	0.0000
Extubation time, min, M ± m	9.89 ± 1.01	11.1 ± 2.31	0.0000
BIS during surgery, CU	43.3 ± 1.86	41.0 ± 1.07	0.4440
BIS after extubation, CU	91.6 ± 2.57	85.7 ± 14.5	0.0000
Grading of pain on the NRS (points):			0.017
0–3	16 (59%)	6 (23%)	
4–7	10 (37%)	15 (58%)	
8–10	1 (4%)	5 (19%)	
Fentanyl consumption, mg	0.550 ± 0.061	0.680 ± 0.080	0.048

**Table 3:** Incidence of postoperative complications according to the Clavien-Dindo grading.

Complications	Xenon + DMM Anesthesia, *n* = 27	Sevoflurane Anesthesia, *n* = 26	*p*-Level
Number of patients with complication	17 (62.9%)	25 (96.2%)	**0.003**
**Clavien-Dindo I**	8 (29.6%)	23 (88.4%)	**0.042**
Postoperative nausea and vomiting	1 (3.7%)	9 (34.6%)	**0.042**
Hyperthermia	5 (18.5%)	12 (46.2%)	0.204
Reactive pleurisy	2 (7.4%)	2 (7.7%)	0.640
**Clavien-Dindo II**	5 (18.5%)	20 (76.9%)	**0.019**
Pulmonary embolism	0	3 (11.5%)	0.261
Anemia	1 (3.7%)	4 (15.4%)	0.393
Pneumonia	2 (7.4%)	3 (11.5%)	1.000
Postoperative psychosis	0	2 (7.7%)	0.488
Postoperative pancreatitis	2 (7.4%)	8 (30.8%)	0.146
**Clavien-Dindo IIIa**			
Inflammation of the postoperative wound	1 (3.7%)	3 (11.5%)	0.630
**Clavien-Dindo IIIb**	3 (11.1%)	8 (30.8%)	0.272
Bleeding	1 (3.7%)	3 (11.5%)	0.630
Anastomotic leak	1 (3.7%)	4 (15.4%)	0.393
Eventration	1 (3.7%)	1 (3.8%)	0.488
**Clavien-Dindo IVa**			
Acute cerebrovascular accident	0	1 (3.8%)	1.000
**Clavien-Dindo IVb**			
Multiple organ failure	1 (3.7%)	3 (11.5%)	0.630
**Clavien-Dindo V**	0	1 (3.8%)	1.000

**Table 4:** Characteristics of variables of the Clavien-Dindo IIIb-V complication prediction model in patients with gastric cancer (variant 1).

Variables	B	Standard Error of the Mean	Wald Coefficient	*p* Level
Type of anesthesia	1.793	1.139	2.477	0.116
sAP	−0.1	0.048	4.323	0.038
CT EXTEM	0.144	0.103	1.950	0.163
Alpha angle INTEM	0.291	0.227	1.641	0.2
Constant	−24.112	17.869	1.821	0.177

**Table 5:** Characteristics of the variables of the model for predicting severe pain syndrome (variant 1).

Variables	B	Standard Error of the Mean	Wald Coefficient	*p* Level
Type of anesthesia	4.961	6.77	0.537	0.464
Glucose	−4.373	3.934	1.235	0.266
CFT EXTEM	0.145	0.124	1.384	0.239
MCF EXTEM	0.129	0.369	0.122	0.727
A (10) INTEM	−0.136	0.178	0.582	0.446
Alpha angle INTEM	−0.43	0.378	1.29	0.256
Constant	33.422	33.631	0.988	0.32

**Table 6:** Characteristics of variables of the Clavien-Dindo IIIb-V complication prediction model in patients with gastric cancer (variant 2).

Variables	B	Standard Error of the Mean	Wald Coefficient	*p* Level
Type of anesthesia	0.874	1.034	0.714	0.398
sBP	−0.2	0.053	0.141	0.708
CFT EXTEM	−0.1	0.023	0.186	0.66
CT INTEM	0.037	0.022	2.866	0.09
Constant	−5.216	5.701	0.837	0.36

**Table 7:** Characteristics of the variables of the model for predicting severe pain syndrome (variant 2).

Variables	B	Standard Error of the Mean	Wald Coefficient	*p* Level
Anesthesia type	0.514	1.59	0.105	0.746
Glucose	0.487	0.314	2.405	0.121
A(10) INTEM	−0.107	0.136	0.611	0.434
SFMC	0.349	0.309	1.271	0.260
Constant	−2.213	9.120	0.059	0.808

## Data Availability

The original contributions presented in this study are included in the article. Further inquiries can be directed to the corresponding author.

## Abbreviations

NRS	numeric rating scale
APTT	activated partial thromboplastin time
ASA	American Society of Anesthesiologists Physical Status Classification System
sBP	systolic blood pressure
SFMC	soluble fibrin-monomer complexes
DMM	Dexmedetomidine
eCCO	estimated continuous cardiac output
eCI	estimated cardiac index
eSV	estimated stroke volume
eSI	estimated stroke index
SpO2	arterial hemoglobin oxygen saturation
IL	Interleukin
ACTH	adrenocorticotropic hormone
ROTEM/EXTEM/INTEM	rotational thromboelastometry and its modifications
CT	the clot formation time required to reach an amplitude of 2 mm, characterizes the initial stage (initiation) of blood coagulation
CFT	clot formation time (the time interval between reaching 2- and 20-mm amplitudes), characterizes fibrin polymerization under the influence of coagulation factor XIII and platelets
A(10)	the clot hardness after 10 min, characterizing the density and stability of the clot at the specified time
MCF	the maximum clot hardness
MCE	the maximum flexibility (elasticity) of the clot
CD11b, CD66b and CD14	markers of neutrophils and monocytes
AMPA, NMDA	glutamate receptors
